# Design and rationale for a pragmatic cluster randomized trial of the Cardiovascular Health Awareness Program (CHAP) for social housing residents in Ontario and Quebec, Canada

**DOI:** 10.1186/s13063-019-3806-5

**Published:** 2019-12-23

**Authors:** Gina Agarwal, Magali Girard, Ricardo Angeles, Melissa Pirrie, Marie-Thérèse Lussier, Francine Marzanek, Lisa Dolovich, J. Michael Paterson, Lehana Thabane, Janusz Kaczorowski

**Affiliations:** 1 Department of Family Medicine, McMaster University, 100 Main Street West, DBHSC, 5th Floor, Hamilton, ON L8P 1H6 Canada; 2 Department of Health Research Methods, Evidence, and Impact, McMaster University, Hamilton, Canada; 3 Centre hospitalier de l’Université de Montréal (CHUM), Montréal, Canada; 4 Department of Family and Emergency Medicine, Université de Montréal, Montreal, Canada; 5 Primary Care Research Team, Centre intégré de santé et des services sociaux de Laval, Laval, QC Canada; 6 Leslie Dan Faculty of Pharmacy, University of Toronto, Toronto, Canada; 7 Institute for Clinical Evaluative Sciences, Toronto, Canada; 8 Institute of Health Policy, Management and Evaluation, University of Toronto, Toronto, Canada; 9 Biostatistics Unit, St Joseph’s Healthcare Research Institute, Hamilton, Canada

**Keywords:** Hypertension, Diabetes, Cardiometabolic risk factors, Poverty, Social housing, Older adults, Volunteer

## Abstract

**Background:**

The Cardiovascular Health Awareness Program (CHAP) uses volunteers to provide cardiovascular disease (CVD) and diabetes screening in a community setting, referrals to primary care providers, and locally available programs targeting lifestyle modification. CHAP has been adapted to target older adults residing in social housing, a vulnerable segment of the population. Older adults living in social housing report poorer health status and have a higher burden of a multitude of chronic illnesses, such as CVD and diabetes. The study objective is to evaluate whether there is a reduction in unplanned CVD-related Emergency Department (ED) visits and hospital admissions among residents of social seniors’ housing buildings receiving the CHAP program for 1 year compared to residents in matched buildings not receiving the program.

**Methods/design:**

This is a pragmatic, cluster randomized controlled trial in community-based social (subsidized) housing buildings in Ontario and Quebec. All residents of 14 matched pairs (intervention/control) of apartment buildings will be included. Buildings with 50–200 apartment units with the majority of residents aged 55+ and a unique postal code are included. All individuals residing within the buildings at the start of the intervention period are included (intention to treat, open cohort). The intervention instrument consists of CHAP screens for high blood pressure using automated blood pressure monitors and for diabetes using the Canadian Diabetes Risk (CANRISK) assessment tool. Monthly drop-in sessions for screening/monitoring are held within a common area of the building. Group health education sessions are also held monthly. Reports are sent to family doctors, and attendees are encouraged to visit their family doctor. The primary outcome measure is monthly CVD-related ED visits and hospitalizations over a 1-year period post randomization. Secondary outcomes are all ED visits, hospitalizations, quality of life, cost-effectiveness, and participant experience.

**Discussion:**

It is anticipated that CVD-related ED visits and hospitalizations will decrease in the intervention buildings. Using the volunteer-led CHAP program, there is significant opportunity to improve the health of older adults in social housing.

**Trial registration:**

ClinicalTrials.gov,NCT03549845. Registered on 15 May 2018. Updated on 21 May 2019.

## Background

This study applies more than 15 years of knowledge accumulated as part of the Cardiovascular Health Awareness Program (CHAP) with the new goal of improving the cardiovascular health of seniors living in subsidized social housing in Ontario and in Quebec. Older adults living in social housing report poorer health status and have a higher burden of a multitude of chronic illnesses, such as cardiovascular disease (CVD) and diabetes, compared to seniors not living in subsidized housing [1–4].

CHAP is a community-based, patient-oriented, and interdisciplinary CVD prevention and management program. The CHAP model of community-based cardiovascular risk assessment and peer education was successfully implemented in pharmacies as a pragmatic clustered randomized controlled trial (RCT) [5] and has been shown to reduce CVD-related hospitalizations without additional costs to the healthcare system [5, 6]. A Canadian Institutes of Health Research (CIHR) team grant funded multiple pilot studies, adapting the CHAP model to other contexts, including interdisciplinary primary care clinics, places of worship, and community centers. The proposed intervention is based on the collective knowledge and best practices gained from all of these studies.

CHAP has mainly been conducted in community pharmacies, but it has been the subject of pilot projects, proof of concept studies, and RCTs in other settings as well [7–19]. Our current work revolves around building on the success of CHAP across Canada. The goal is to identify optimal conditions for scaling up and leveraging resources within communities to enable CHAP to become an ongoing initiative available to all Canadians to significantly improve community and population-based prevention and management of CVD. Although CHAP has been the subject of several studies in community pharmacies, strong clinical and policy recommendations are inevitably based on results of several high-quality RCTs conducted in different settings and populations that cumulatively have shown the benefit of the intervention in question. Therefore, a trial is needed now, to assess whether similar results can be replicated in a different context that targets a different population using a modified intervention. Based upon the promising findings from an existing community health program in subsidized housing involving specially trained paramedics, it is apparent that the CHAP model using volunteers could potentially be successful in this context [20, 21]. A full-scale RCT of the intervention is warranted. This project focuses on a more vulnerable segment of the older adult population, those living in social housing [21], compared to the typical participants in our pharmacy-based trial.

This adaptation of the CHAP program will include, for the first time, group-based educational sessions aimed at increasing cardiovascular health awareness to promote healthy habits and self-management among residents. Also, this project will assess the impact of less intense, but regular, year-round screening and monitoring sessions, which may be easier to integrate within social housing and to sustain over time.

## Methods/design

### Study aims

This study aims to determine whether there is a relative reduction in the composite outcome of unplanned *CVD-related Emergency Department (ED) or hospital admission* among residents of subsidized seniors’ housing buildings receiving the CHAP program for 1 year compared to residents in matched buildings not receiving the program.

In addition, it aims to determine:

- The difference in *unplanned, all-cause healthcare utilization* (as measured by unplanned, all-cause ED visits and non-elective, all-cause hospitalization rates) among residents of subsidized housing buildings receiving the CHAP program for 1 year compared to those in matched buildings not receiving the program
- The difference in *quality of life and quality-adjusted life years (QALYs)* as measured by the EuroQoL five-dimension, five-level (EQ-5D-5L) instrument in participants attending the CHAP sessions over 1 year, compared to their baseline measure
- The *cost-effectiveness* of the program
- *Participant, volunteer, and community relations workers' experiences* regarding the program
- Relative changes in *risk factor measures* (such as blood pressure [BP], body mass index [BMI], and diabetes risk status) in participants attending the CHAP sessions compared to their baseline measurements.

### Study design

**Fig. 1:**
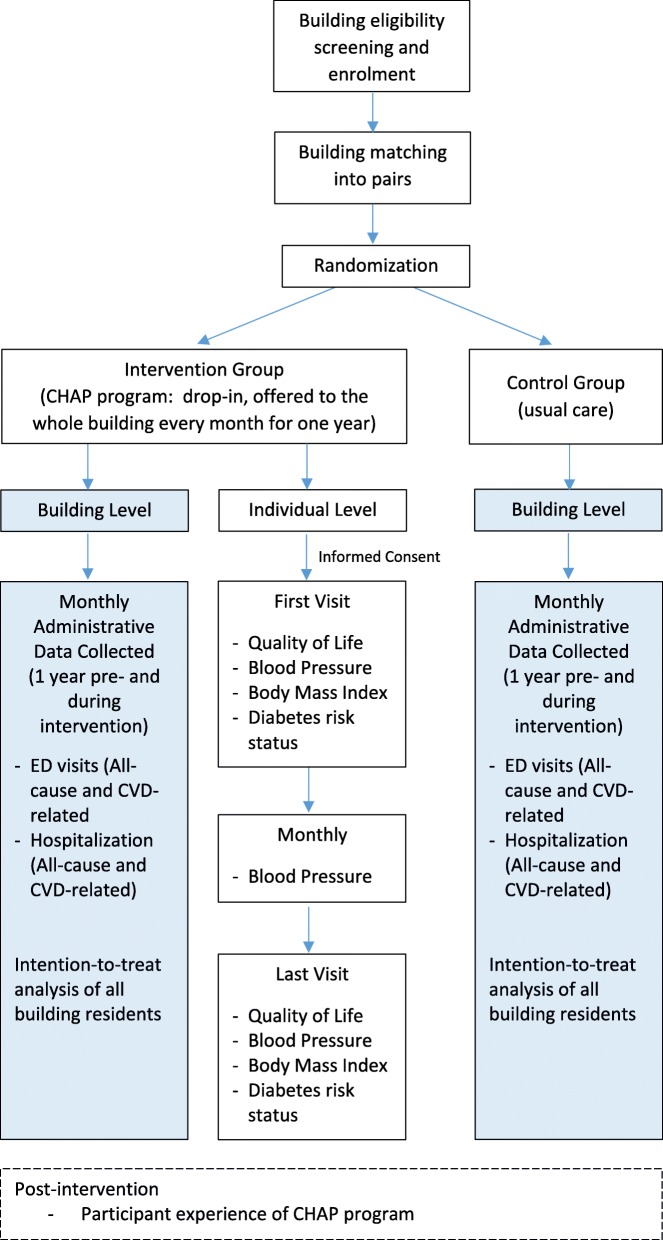
Study design flowchart

**Fig. 2:**
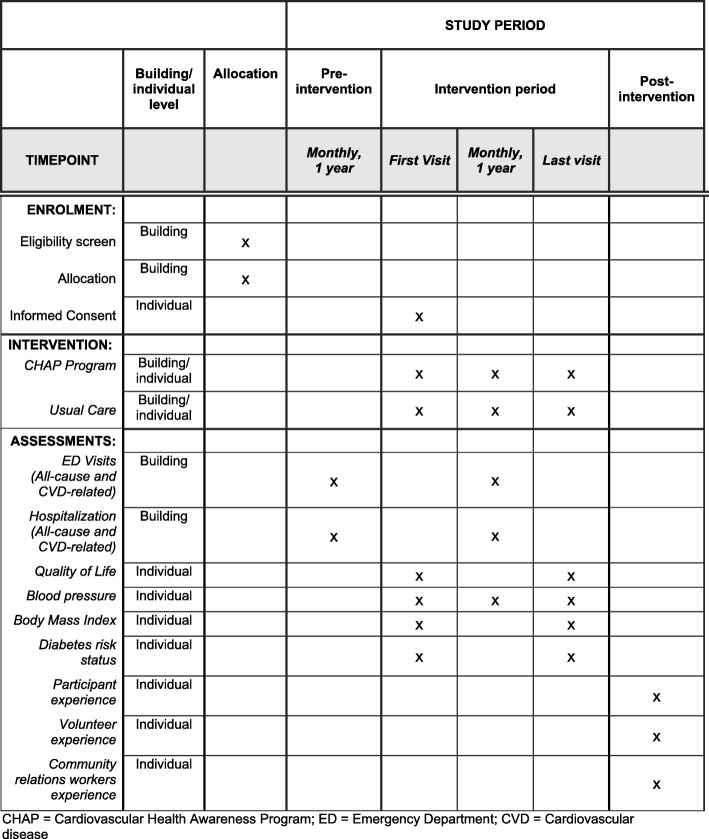
Standard Protocol Items: Recommendations for Interventional Trials (SPIRIT) figure showing schedule of interventions and assessments. *CHAP* Cardiovascular Health Awareness Program, *ED* Emergency Department, *CVD* cardiovascular disease

The trial will be an open-label, parallel, cluster RCT and is being reported according to the Standard Protocol Items: Recommendations for Interventional Trials (SPIRIT) checklist (see Additional file 1). Buildings will be the primary unit of analysis. It will be an open cohort study with repeated cross-sectional assessments comparing the outcomes of intervention and control buildings during the pre-intervention and 1-year post-intervention periods. See Fig. 1 for the study flowchart and Fig. 2 for the SPIRIT study schedule.

The trial will take place in the provinces of Ontario and Quebec, in two selected regions. Pairs (intervention-control) of subsidized seniors’ housing buildings will be selected from each province with input from local social housing provider/organization partners.

### Inclusion and exclusion criteria

Designated seniors’ buildings with 50–200 apartment units and a majority of residents aged 55 years or older will be included in the study. The buildings will have a unique postal code to be included in the sample. There are no exclusion criteria. All residents living in the building during the pre-intervention and post-intervention periods will be taken as samples for intervention and control buildings.

### Recruitment process

Recruitment of buildings will be through regions or localities in each province that have similar characteristics. Social housing organizations will be contacted and introduced to the CHAP program. Leading up to the trial implementation, presentations to managers and chief executive officers of housing organizations will be made. Previous work by the McMaster Community Paramedicine (MCP) research team has shown that it has been possible to recruit multiple social housing buildings in five separate localities across Ontario for participation in an RCT [22]; a total of 32 seniors’ housing buildings with an average of 126 apartment units each were recruited. We anticipate that we will be able to recruit the necessary number of buildings from each province (100% recruitment rate). Residents, volunteers, and housing staff of intervention buildings in each province will be recruited for four focus groups via posters and advertisements following the last CHAP session.

### Sample size

For the main outcome (ED visits and hospitalization) we computed the sample size based on the number of ambulatory care-sensitive ED visits from Canadian Institute for Health Information (CIHI) data [23]. The estimated mean number of ED visits for an individual is 0.39 (standard deviation [SD] = 0.12). We assumed that the intervention will lead to 9% difference in the mean number of ED visits by residents of intervention buildings compared to control building residents. This was based on the effect CHAP had on the hospitalization rates in the community cluster RCT [5]. We also assumed an intraclass correlation coefficient (ICC) of 0.07 for our design effect based on data analyzed from previous studies [21, 24]. Using a power of 0.8 and an alpha of 0.05, we will need a total of 1118 subsidized housing residents for each arm of the study. This is the population of approximately 12 subsidized housing buildings. We will therefore require a minimum of 12 pairs (intervention-control) of buildings; 6 pairs will be in Ontario and 6 will be in Quebec. This number is feasible based on our pre-implementation discussions with the housing providers in both provinces.

### Allocation of intervention

Buildings will be organized into comparable pairs based on geographic, resource, and demographic characteristics. Each building pair will be allocated to receive or not receive the intervention (1:1 ratio) via computer-generated paired randomization by a statistician not affiliated with the project.

### Assessment measures

#### Primary and secondary outcome measures

For the composite primary outcome, we will compare the healthcare utilization of intervention and control buildings as measured by:

1. Change in the monthly rate of CVD-related ED visits from 1 year pre intervention to 1 year post intervention
2. Change in the monthly rate of CVD-related hospitalization from 1 year pre intervention to 1 year post intervention.

Secondary outcome measures will include:

1. Difference in unplanned all-cause healthcare utilization, measured by unplanned all-cause ED visit rates and unplanned all-cause hospitalization rates between intervention and control group from 1 year pre intervention to 1 year post intervention
2. Difference in the quality of life and QALYs (measured by the EQ-5D-5L) in CHAP participants from baseline to 1 year post intervention
3. Cost-effectiveness, based on program cost of implementing CHAP for 1 year in subsidized seniors’ housing buildings and measured outcomes
4. Participants’ experience of the CHAP intervention (qualitative).

Other pre-specified outcome measures include:

1. Change in BP among CHAP participants from baseline to 1 year post intervention
2. Change in BMI from baseline to 1 year post intervention
3. Change in waist circumference from baseline to 1 year post intervention
4. Change in Canadian Diabetes Risk (CANRISK) Questionnaire score measured using the baseline to 1year post intervention.

#### Process evaluation measures

Process evaluation measures will include resident participation rates (number of participants attending CHAP sessions, number of participants attending education sessions, including initial attendance and repeat visits), information on volunteers (number trained, number of sessions volunteered), program delivery (e.g., completion of risk assessments), and other program evaluation measures (e.g., detection rates for hypertension and diabetes, number of feedback reports sent to family doctors).

Key informant interviews or focus groups will be conducted post intervention with the CHAP volunteers, housing authorities, participants, and non-participants regarding strengths, weaknesses, and opportunities to improve and sustain the program.

### Intervention

The CHAP sessions will be voluntary drop-in clinics conducted by trained volunteers following pre-specified protocols and supervised by a research nurse to ensure intervention fidelity. Volunteers will be trained in person by CHAP research staff on research processes (privacy/confidentiality, obtaining informed consent), participant assessment, data collection, and use of algorithms to refer participants to the health nurse (see Additional files 2 and 3). The sessions will be delivered in a common space within each intervention building. Sessions will include:

- BP readings using a validated automated BP machine during each monthly CHAP session
- The validated CANRISK diabetes risk questionnaire [25], which measures cardiometabolic risk factors such as BMI, waist circumference, diabetes history, smoking, diet, physical activity, and smoking
- Healthy lifestyle and preventive care materials (e.g., pamphlets)
- Invitation to participate in group health education following each monthly CHAP session. The health education sessions will include common health-related topics and will be delivered by local expert community organizations. The health education is not a standardized program (pragmatic), and it is expected that there will be heterogeneity between communities and buildings
- Upon participant’s consent, blood pressure, BMI, waist circumference and CANRISK score sent to family doctor.

All tenants in intervention buildings will be invited to participate in CHAP sessions and subsequent education sessions using multiple recruitment strategies, including posters in common areas. Participation is voluntary, and all participants will provide written informed consent for their individual-level data to be collected (see Additional file 4).

The control buildings will receive usual care, which will be any wellness programs if already present in the building prior to the RCT. Not all control buildings will have wellness programs.

### Data collection

Data during the CHAP intervention session will be collected either on paper or through an electronic form. All data collected will be protected and kept confidential. Paper forms will be kept in locked cabinets in the research office. All data in the electronic platform will be kept in encrypted and firewall-protected servers only accessible to the research team, volunteers, and nurses implementing the program. All data analysis of collected data will be anonymized.

The primary outcomes will be assessed using de-identified administrative datasets in Ontario and Quebec. We will use building-level data for each of these outcomes, at monthly intervals for 1 year prior to and for the 1-year duration of the program.

Quality of life data will be collected using the EQ-5D-5 L, administered by a research assistant or volunteer to all residents of intervention buildings who attend a CHAP session. This will be done at two time points, when the participants attend their first CHAP session and then at the final CHAP session. Cost-effectiveness will be calculated by collection of program cost data and health outcomes. Health experiences and perceptions of participants will be collected at the end of the study by a trained research assistant using four qualitative focus groups of intervention residents, including two in Ontario and two in Quebec.

### Data analysis plan

#### Quantitative data

We will analyze data with the building or cluster as the primary unit of analysis, rather than the individual. The reason is that it is not possible to acquire individual-level consent from the control building residents to obtain their health administrative data. It is possible to get this information in aggregate form for each building.

The analysis and reporting of the trial will follow the Consolidated Standards of Reporting Trials (CONSORT) extension to cluster trials [26]. The analysis of the baseline characteristics will be based on descriptive statistics reported as count (percent) for categorical variables and mean (SD) or median (first quartile, third quartile) for continuous variables. The analysis of primary and secondary outcomes will follow the intention-to-treat principle. For the primary building-level outcome measure we will analyze the data using analysis of covariance (ANCOVA), adjusting for pairing (fixed-effect) and baseline rate (covariate) in the model. For the secondary individual-level data (quality of life, QALY), we will use the intention-to-treat principle analysis and analyze the data using generalized estimating equations (GEEs) assuming an exchangeable correlation structure to adjust for clustering within a building [27]. Multiple imputation (iterative Markov chain Monte Carlo method) with five imputations will be used if more than 5% of the participant outcome data is missing in the follow-up survey [28]. All analyses will be performed using STATA 11.2.

Since CHAP is a health promotion program that can be integrated into the health system and is expected to impact health system utilization, we will conduct a cost-effectiveness analysis. This analysis will be conducted at a building level and will therefore be based on administrative data and program costs. Therefore, the sample size will be the same as that for the main outcome.

#### Qualitative data

We will use a grounded theory approach. We will start with open coding followed by axial coding and theory generation to explain the process experience by participants as they attend the CHAP intervention.

#### Other analyses

We will conduct a quarterly process analysis in order to maintain trial fidelity (described earlier under Process evaluation measures). The main analyses will take place at the end of the trial. A subgroup analysis by gender is planned based on the data that we have available. Regression analysis will be used to determine if gender is a factor affecting our main outcomes.

### Control of bias and loss to follow-up

This will be an open trial with no blinding or masking. Due to the nature of the health intervention (health sessions in intervention buildings), it will not be possible to blind participants as to whether they are receiving the intervention or not. However, since randomization is at the building level and the primary outcome (ED visit rates and hospital admission rates) is at a cluster level and collected from administrative data sources, the study will be less prone to social desirability bias. No consent for the primary outcome will be collected from the intervention and control building residents since it will be based on de-identified administrative data. Consent will only be collected from building residents who attend the CHAP sessions for their participation in the CHAP sessions.

Since the primary outcome is being analyzed at the building level, we anticipate no loss to follow-up for healthcare utilization data. We will retrospectively assess residents who lived in the intervention and control buildings at the pre- and post-intervention periods. There may be movement of residents in and out of the buildings; however, this will likely happen in both intervention and control buildings. This is expected to be balanced between the intervention and control arms. For the secondary outcome measures that require collection of data directly from residents, loss to follow-up is anticipated to be 40% [24]. A previous study conducted in this subsidized housing population found that there was a higher rate of deaths, relocations, and inability to contact in this population compared to the general population [21, 24]. However, we assume that losses to follow-up will be similar between the intervention and control groups.

### Ethical approval

Ethical approval has been obtained from the Hamilton Integrated Research Ethics Board (Project number 5051) and the University of Montreal Hospital Centre (CHUM) Ethics Board (Project number 18.120). Any future modifications to the protocol will be submitted to the respective ethics boards for review through the amendment process. The study is a minimal-risk pragmatic trial; therefore, no data monitoring committee is required.

## Discussion

The intervention we are studying in this trial is an adaptation of the intervention studied in the original pharmacy-based CHAP [5]. Older adults in subsidized housing are a hard-to-reach population with poorer health compared to the general population. This population also has a high prevalence of mobility issues [1]; therefore, conducting CHAP sessions in the common spaces within each building may help overcome this barrier and improve access to cardiometabolic risk assessment and health education. Therefore, it is expected that by providing CHAP in this setting, there will be a similar or greater impact on healthcare utilization and health outcomes, compared to the original CHAP trial in pharmacies [5].

The core elements of CHAP fit well with this population and context. There is minimal equipment or training required, the sessions can be run by volunteers with oversight from a nurse, and they can be held within the common area of each residential building. CHAP also “closes the loop” by encouraging attendees to connect with their family doctor and share their assessment results.

Since this population is naturally clustered within the subsidized housing buildings, implementing a cluster RCT was a natural fit for the study. This new setting also allowed for the addition of pragmatic education sessions; we acknowledge that there will be heterogeneity and it will be difficult to replicate this component. The results of this study will provide evidence on the impact of CHAP in this setting. Decreasing healthcare utilization for CVD-related causes will have an impact on health system and associated costs. These findings will inform the expansion of CHAP throughout each province and across Canada.

Adapting and evaluating the effectiveness of CHAP in social housing will enable it to have a substantial positive impact.. We anticipate improved collaboration between housing authorities, the regional public health, family physicians, community organizations, and training of a new cohort of health educators. Trial results will be disseminated through academic publications and presentations to stakeholders.

These types of changes are called for by major Canadian and American initiatives such as the CIHR’s Strategy for Patient-Oriented Research (SPOR) or the Patient-Centered Outcomes Research Institute (PCORI) in the USA. CHAP has the potential to enhance access and increase capacity of the healthcare system, promote greater coordination of care and integration with community resources, improve quality of care, promote greater involvement of patients in self-care, and, ultimately, reduce morbidity and mortality associated with CVD.

## Supplementary information

**Additional file 1.** SPIRIT 2013 checklist: recommended items to address in a clinical trial protocol and related documents.

**Additional file 2.** Algorithm for blood pressure.

**Additional file 3.** Algorithm for diabetes.

**Additional file 4.** CHAP participant information and informed consent sheet.

## Data Availability

The datasets generated and/or analyzed during the current study are available from the corresponding author on reasonable request. The administrative dataset from this study is held securely in coded form. While data sharing agreements prohibit data holders from making the dataset publicly available, access may be granted to those who meet pre-specified criteria for confidential access. The full dataset creation plan and underlying analytic code are available from the authors upon request, understanding that the programs may rely upon coding templates or macros that are unique to the database holders.

## Abbreviations

BMI: Body mass index
BP: Blood pressure
CANRISK: Canadian Diabetes Risk (Questionnaire)
CHAP: Cardiovascular Health Awareness Program
CIHI: Canadian Institute for Health Information
CIHR: Canadian Institutes of Health Research
CVD: Cardiovascular disease
ED: Emergency Department
GEE: Generalized estimating equation
PCORI: Patient-Centered Outcomes Research Institute
QALY: Quality-adjusted life year
RCT: Randomized controlled trial
SPOR: Strategy for Patient-Oriented Research

## References

[1] Gibler KM (2003). Aging subsidized housing residents: a growing problem in U.S cities. J Real Estate Res.

[2] Rivera LA, Lebenbaum M, Rosella LC (2015). The influence of socioeconomic status on future risk for developing Type 2 diabetes in the Canadian population between 2011 and 2022: differential associations by sex. Int J Equity Health.

[3] Clark AM, DesMeules M, Luo W, Duncan AS, Wielgosz A (2009). Socioeconomic status and cardiovascular disease: risks and implications for care. Nat Rev Cardiol.

[4] Société d’habitation du Québec (2015). La santé des résidents d’HLM: analyse comparative de la santé de la population à faible revenu selon le mode d’occupation.

[5] Kaczorowski J, Chambers LW, Dolovich L, Paterson JM, Karwalajtys T, Gierman T, Sabaldt RJ (2011). Improving cardiovascular health at population level: 39 community cluster randomised trial of Cardiovascular Health Awareness Program (CHAP). BMJ.

[6] Goeree R, Von Keyserlingk C, Burke N, He J, Kaczorowski J, Chambers L, Zagorski B (2013). Economic appraisal of a community-wide cardiovascular health awareness program. Value Health.

[7] Karwalajtys T, Kaczorowski J, Chambers LW, Levitt C, Dolovich L, McDonough B, Williams JE (2005). A randomized trial of mail vs. telephone invitation to a community-based cardiovascular health awareness program for older family practice patients. BMC Fam Pract.

[8] Chambers LW, Kaczorowski J, Dolovich L, Karwalajtys T, Hall HL, McDonough B, Levitt C (2005). A community-based program for cardiovascular health awareness. Can J Public Health/Revue Canadienne de Santé Publique.

[9] Pora VV, Farrell B, Dolovich L, Kaczorowski J, Chambers L (2005). Promoting cardiovascular health among older adults: a pilot study with community pharmacists contributed to the design of a multidisciplinary approach to conducting blood pressure sessions in community pharmacies. Can Pharm J/ Revue des Pharmaciens du Canada.

[10] Lau E, Kaczorowski J, Karwalajtys T, Dolovich L, Levine M, Chambers L (2006). Blood pressure awareness and self-monitoring practices among primary care elderly patients. Can Pharm J/Revue des Pharmaciens du Canada.

[11] Broomfield J, Schieda N, Sullivan SM, Chambers LW, Kaczorowski J, Karwalajtys T (2008). Recording blood pressure readings in elderly patients’ charts: what patient and physician characteristics make it more likely?. Can Fam Physician/Médecin de Famille Canadien.

[12] Karwalajtys T, McDonough B, Hall H, Guirguis-Younger M, Chambers LW, Kaczorowski J, Hutchison B (2009). Development of the volunteer peer educator role in a community Cardiovascular Health Awareness Program (CHAP): a process evaluation in two communities. J Community Health.

[13] Carter M, Karwalajtys T, Chambers L, Kaczorowski J, Dolovich L, Gierman T, Laryea S (2009). Implementing a standardized community-based cardiovascular risk assessment program in 20 Ontario communities. Health Promot Int.

[14] Angeles RN, Dolovich L, Kaczorowski J, Thabane L (2014). Developing a theoretical framework for complex community-based interventions. Health Promot Pract.

[15] Chambers LW, Kaczorowsk J, O’Rielly S, Ignagni S, Hearps SJC (2013). Comparison of blood pressure measurements using an automated blood pressure device in community pharmacies and family physicians’ offices: a randomized controlled trial. CMAJ Open.

[16] Jones CA, Nanji A, Mawani S, Davachi S, Ross L, Vollman A, Campbell N (2013). Feasibility of community-based screening for cardiovascular disease risk in an ethnic community: the South Asian Cardiovascular Health Assessment and Management Program (SA-CHAMP). BMC Public Health.

[17] Karwalajtys T, Kaczorowski J, Chambers LW, Hall H, McDonough B, Dolovich L, Hutchison B (2013). Community mobilization, participation, and blood pressure status in a Cardiovascular Health Awareness Program in Ontario. Am J Health Promot.

[18] Thabane L, Kaczorowski J, Dolovich L, Chambers LW, Mbuagbaw L, CHAP Investigators (2015). Reducing the confusion and controversies around pragmatic trials: using the Cardiovascular Health Awareness Program (CHAP) trial as an illustrative example. Trials.

[19] Myers MG, Kaczorowski J, Paterson JM, Dolovich L, Tu K (2015). Thresholds for diagnosing hypertension based on automated office blood pressure measurements and cardiovascular risk. Hypertension.

[20] Agarwal G, Angeles R, Pirrie M, Marzenek F, McLeod B, Parascandalo J, Dolovich L (2017). Effectiveness of a community paramedic-led health assessment and education initiative in a seniors’ residence building: the Community Health Assessment Program through Emergency Medical Services (CHAP-EMS). BMC Emerg Med.

[21] Agarwal Gina, Angeles Ricardo, Pirrie Melissa, McLeod Brent, Marzanek Francine, Parascandalo Jenna, Thabane Lehana (2019). Reducing 9-1-1 Emergency Medical Service Calls By Implementing A Community Paramedicine Program For Vulnerable Older Adults In Public Housing In Canada: A Multi-Site Cluster Randomized Controlled Trial. Prehospital Emergency Care.

[22] Agarwal G, McDonough B, Angeles R, Pirrie M, Marzanek F, McLeod B, Dolovich L (2015). Rationale and methods of a multicentre randomised controlled trial of the effectiveness of a Community Health Assessment Programme with Emergency Medical Services (CHAP-EMS) implemented on residents aged 55 years and older in subsidised seniors’ housing buildings in Ontario, Canada. BMJ Open.

[23] Canadian Institute for Health Information (CIHI) (2014). Pan-Canadian Forum for High Users of Health Care.

[24] Agarwal G, Angeles R, Pirrie M, McLeod B, Marzanek F, Parascandalo J, Thabane L (2018). Evaluation of a community paramedicine health promotion and lifestyle risk assessment program for older adults who live in social housing: a cluster randomized trial. CMAJ.

[25] Robinson CA, Agarwal G, Nerenberg K (2011). Validating the CANRISK prognostic model for assessing diabetes risk in Canada’s multi-ethnic population. Chronic Dis Inj Can.

[26] Campbell MK, Elbourne DR, Altman DG (2004). CONSORT statement: extension to cluster randomised trials. BMJ.

[27] Norton EC, Bieler GS, Ennett ST, Zarkin GA (1996). Analysis of prevention program effectiveness with clustered data using generalized estimating equations. J Consult Clin Psychol.

[28] Jakobsen JC, Gluud C, Wetterslev J, Winkel P (2017). When and how should multiple imputation be used for handling missing data in randomised clinical trials-a practical guide with flowcharts. BMC Med Res Methodol.

