# Supplementary material for: Design and rationale for a pragmatic cluster randomized trial of the Cardiovascular Health Awareness Program (CHAP) for social housing residents in Ontario and Quebec, Canada
Source: Trials. 2019 Dec 23;20:760. doi: 10.1186/s13063-019-3806-5 (PMC6929306; doi:10.1186/s13063-019-3806-5)

Additional file 2

Table S1: Blood Pressure Algorithm

| Assessment Area | Test/Tool | Brief Description | Algorithm/Scoring | Next Step |
| --- | --- | --- | --- | --- |
| Blood Pressure | CHAP Algorithm |  | Anything above 140-159 (Systolic BP) and 90-99 (Diastolic BP)→ Positive Screen | Positive Screen → Follow Algorithm below |

Figure S1: Blood Pressure Algorithm
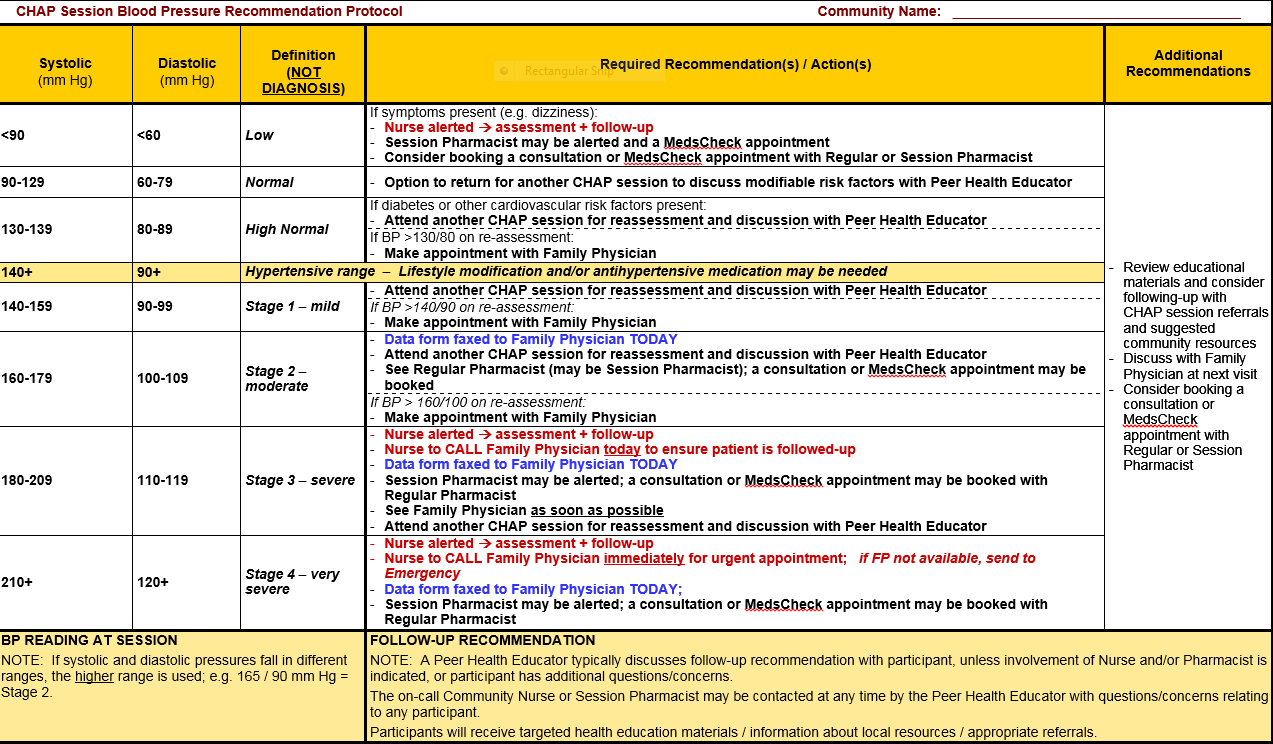

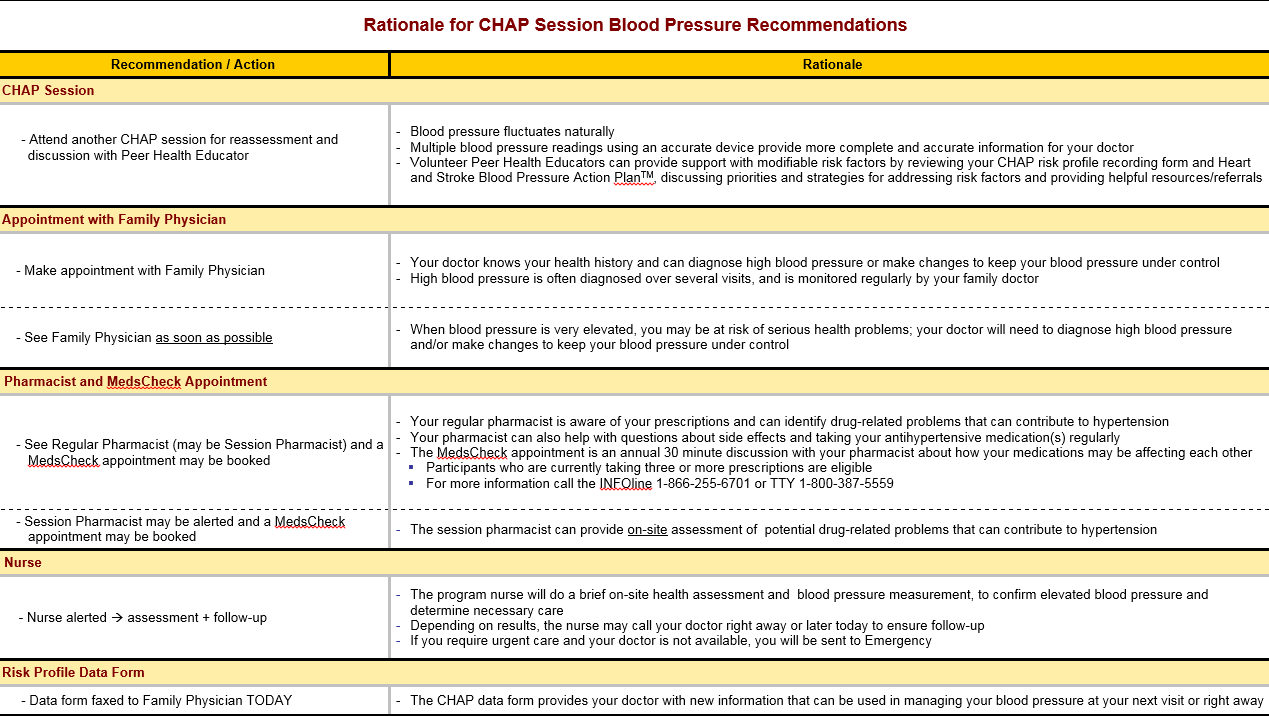

Supplement: Supplementary file 2 — Additional file 2. Algorithm for blood pressure. [file 13063_2019_3806_MOESM2_ESM.docx]
