# Supplementary material for: Design and rationale for a pragmatic cluster randomized trial of the Cardiovascular Health Awareness Program (CHAP) for social housing residents in Ontario and Quebec, Canada
Source: Trials. 2019 Dec 23;20:760. doi: 10.1186/s13063-019-3806-5 (PMC6929306; doi:10.1186/s13063-019-3806-5)

Additional file 3

Table S2: Diabetes Algorithm (CANRISK)

| Assessment Area | Test/Tool | Brief Description | Algorithm/Scoring | Next Step |
| --- | --- | --- | --- | --- |
| Diabetes (If participant is not known to have diabetes) | CANRISK Tool | Canadian Diabetes Risk Questionnaire (PHAC) | Score of 21 or less→ Low Risk (Negative Screen)  Score of 21-32→ Moderate Risk (Positive Screen)  Score of 33 and over→ High Risk (Positive Screen) | Moderate Risk Positive Screen →  Suggest attendance at diabetes education session or Family Physician  High Risk Positive Screen → 1)Inform research Nurse 2)Refer to Family Physicians 3)Suggest attendance at diabetes education session |

CANRISK Questionnaire
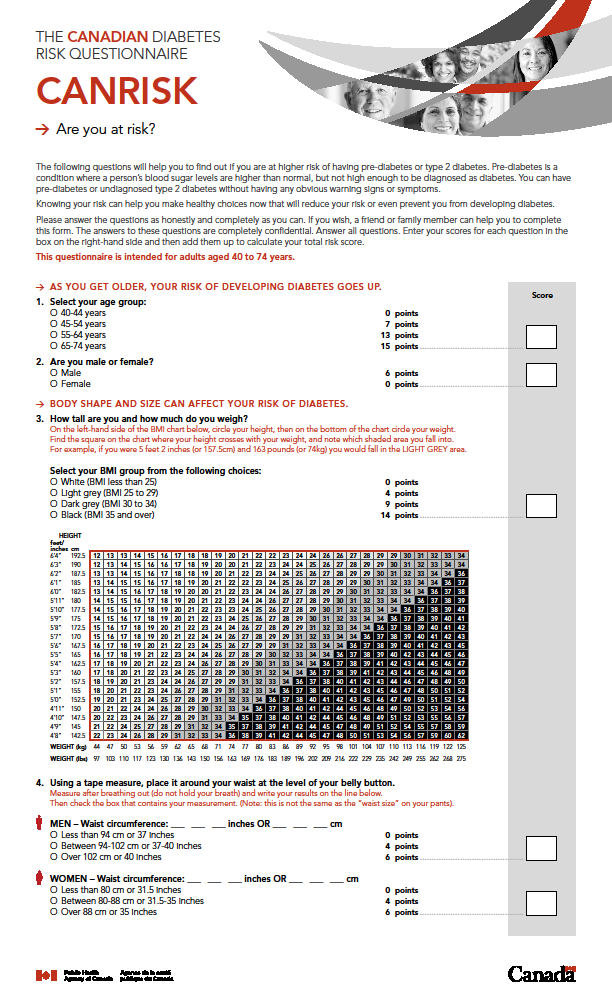


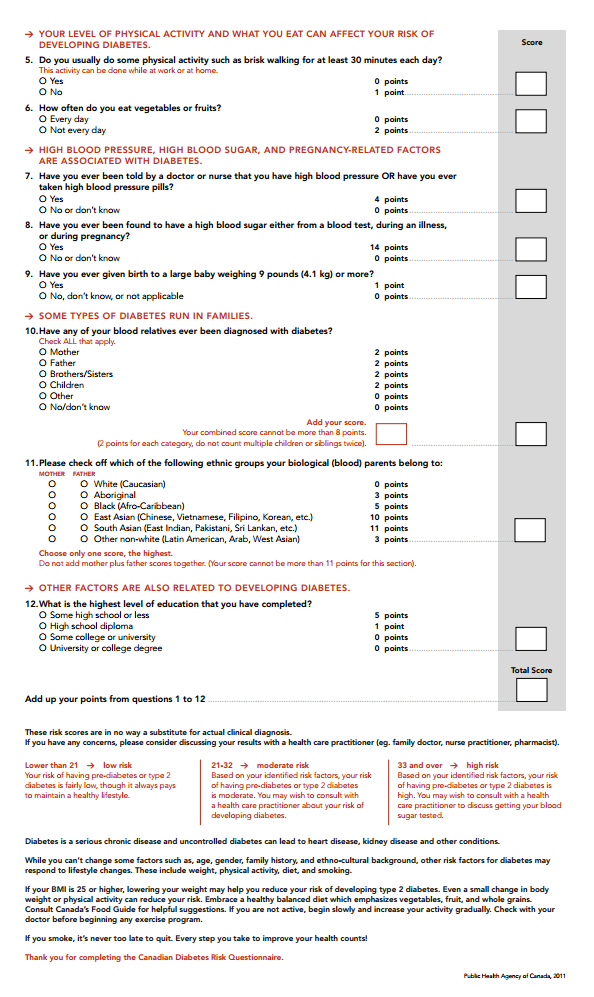

Supplement: Supplementary file 3 — Additional file 3. Algorithm for diabetes. [file 13063_2019_3806_MOESM3_ESM.docx]
