# Supplementary material for: Design and rationale for a pragmatic cluster randomized trial of the Cardiovascular Health Awareness Program (CHAP) for social housing residents in Ontario and Quebec, Canada
Source: Trials. 2019 Dec 23;20:760. doi: 10.1186/s13063-019-3806-5 (PMC6929306; doi:10.1186/s13063-019-3806-5)
Supplement: Supplementary file 4 — Additional file 4. CHAP participant information and informed consent sheet. [file 13063_2019_3806_MOESM4_ESM.doc]

**Participant Information Sheet**

**Project Title:** Pragmatic cluster randomized controlled trial of the Cardiovascular Health Awareness Program (CHAP) in subsidized social housing

**Principal Investigator:** Dr. Gina Agarwal, Department of Family Medicine, McMaster University

**Funding Sponsor:** Canadian Institutes of Health Research

**________________________________________________________________________________**

**Why is this research being done?**

You are being invited to participate in a research study conducted by Dr. Gina Agarwal because you are a resident of a seniors housing complex. This study will help us to find out if the Cardiovascular Health Awareness Program (CHAP) helps improve the health of older adults leading to less emergency and hospital visits.

In order to decide whether or not you want to be a part of this research study, you should understand what is involved and the potential risks and benefits. This form gives detailed information about the research study, which will be discussed with you. Once you understand the study, you will be asked to sign this form if you wish to participate. Please take your time to make your decision. Feel free to discuss it with your friends and family.

**What is the CHAP Program?**

The Cardiovascular Health Awareness Program (CHAP) is a way to have your blood pressure measured by a trained volunteer with an accurate automated blood pressure machine and to complete a checklist of other risk factors for heart disease, stroke and diabetes. Your results can be sent directly to your family doctor with your consent. You can give the name of your doctor or primary care provider when completing your risk profile form. Your heart disease, stroke and diabetes risk profile can help your family doctor determine whether you should be monitored more often to see whether you have high blood pressure, or are at risk for other health problems. If you are already being treated for high blood pressure, the information helps your family doctor to know whether your blood pressure is under control.

**What will my responsibilities be if I take part in the study?**

Participation involves having your blood pressure measured by a trained volunteer using an automated blood pressure machine, completing a heart disease and stroke risk factor profile and diabetes risk assessment. You will be given a copy of your results. You can talk to your family doctor if you have questions or concerns.

It’s a good idea to go to more than one CHAP session to help you identify and manage your risk factors like healthy food choices and level of physical activity. You will also be able to attend a health education session after each CHAP session in your building. You can choose whether or not to participate in any activity.

Your decision to participate or not in **any** aspect of the program will **not** affect the care that you receive from any health professional.

**Why is this Study Being Done?**

High blood pressure and diabetes can increase your risk of getting heart disease, kidney disease, or Alzheimer’s disease, and of having a stroke. It is important to have your blood pressure and risk for diabetes checked regularly, since high blood pressure often has no warning signs or symptoms.

Regular monitoring can help ensure that high blood pressure and diabetes are diagnosed before they lead to other health problems. If you are already being treated for high blood pressure, monitoring helps to make sure that the treatment is working to keep your blood pressure under control. Blood pressure readings from CHAP sessions can help your family doctor know what your blood pressure is when you are not in the doctor’s office. A diabetes assessment from CHAP can help your family doctor decide whether you need to be screened or treated for diabetes.

Assessment for these problems on a regular basis can help to reduce your need to visit the emergency department for serious health problems, by preventing some of these problems before they develop.

**Who is doing this study?**

Researchers from McMaster University, Department of Family Medicine in partnership with your Niagara Regional Housing.

**What are the possible benefits for me and/or society?**

The effect of this program on cardiovascular health is being studied in those residents participating from seniors’ buildings. CHAP participants will be able to go to learn about risk factors for high blood pressure and diabetes.

**What are the Possible Risks and Discomforts?**

There are no anticipated risks associated with study participation. It is possible that during the CHAP session, you may feel uncomfortable sharing your information. You can stop participating at anytime if you feel uncomfortable. It is important for you to know that you can choose not to take part in the study. You are free to withdraw from this study at any time without consequence.

**What information will be kept private?**

Your data will not be shared with anyone except with your consent or as required by law. All personal information such as your name, address, phone number, will be removed from the data and will be replaced with a number. A list linking the number with your name will be kept in a secure place, separate from your file. The data, with identifying information removed will be securely stored in a locked office in the Department of Family Medicine, McMaster University.

Your identifying information (name, gender, date of birth and postal code) and your blood pressure and cardiovascular risk information will be linked to Ontario Ministry of Health and Long-Term Care health care records, such as doctor billings and hospital visit records to study the effects of the CHAP program. Once this information is linked, your identifying information will be removed so that your records are anonymized and no longer identified with you. This research involving health care records will be done at the Institute for Clinical Evaluative Sciences (ICES), an independent, non-profit research organization that has special ‘Prescribed Entity’ status under Ontario’s Privacy legislation. This means ICES is permitted to do research using personal health information and is held to rigorous standards for data security and privacy protection by the Information and Privacy Commissioner of Ontario.

For the purposes of ensuring the proper monitoring of the research study, it is possible that a member of the Hamilton Integrated Research Ethics Board and representatives of the Department of Family Medicine may consult your research data. However, no records which identify you by name or initials will be allowed to leave the Department of Family Medicine, McMaster University offices. By signing this consent form, you or your legally acceptable representative authorizes such access.

If the results of the study are published, your name will not be used and no information that discloses your identity will be released or published without your specific consent to do so.

**Can Participation in the Study End Early?**

If you volunteer to be in this study, you may withdraw at any time. You have the option of removing your data from the study. You may also refuse to answer any questions you don’t want to answer and still remain in the study. The investigator may withdraw you from this research if circumstances arise which warrant doing so.

**Are there any Costs to the Study?**

There is no cost to attending the CHAP sessions. They are available free of charge.

**If I have any Questions or Problems, Who do I Call?**

If you have any questions or concerns, please call the Research Coordinator, Francine Marzanek at

(905) 525-9140, ext. 28501. You can also ask questions of the Principal Investigator of the study Dr. Gina Agarwal, McMaster University, at 905-525-9140 ext. 28520

**CONSENT STATEMENT**

**Participant:**

I have read the preceding information thoroughly. I have had an opportunity to ask questions and all of my questions have been answered to my satisfaction. I agree to participate in this study. I understand that I will receive a signed copy of this form.

- I also agree to have my CHAP session results sent to my primary care provider. I can identify my primary care provider when participating in the CHAP session.

**____________________________________________________________________________________**

**Name Signature Date**

**Person obtaining consent**:

**I have discussed this study in detail with the participant. I believe the participant understands what is involved in this study.**

| **Name, Role in Study** | **Signature** | **Date** |
| --- | --- | --- |

This study has been reviewed by the Hamilton Integrated Research Ethics Board (HIREB). The HIREB is responsible for ensuring that participants are informed of the risks associated with the research, and that participants are free to decide if participation is right for them. If you have any questions about your rights as a research participant, please call the Office of the Chair, Hamilton Integrated

Research Ethics Board at 905.521.2100 x 42013.
